# Identification and validation of a 17-gene signature to improve the survival prediction of gliomas

**DOI:** 10.3389/fimmu.2022.1000396

**Published:** 2022-09-29

**Authors:** Shiao Tong, Minqi Xia, Yang Xu, Qian Sun, Liguo Ye, Jiayang Cai, Zhang Ye, Daofeng Tian

**Affiliations:** ^1^ Department of Neurosurgery, Renmin Hospital of Wuhan University, Wuhan, China; ^2^ Department of Endocrinology & Metabolism, Renmin Hospital of Wuhan University, Wuhan, China

**Keywords:** glioma, prognosis, gene signature, rick score, immunity

## Abstract

Gliomas are one of the most frequent types of nervous system tumours and have significant morbidity and mortality rates. As a result, it is critical to fully comprehend the molecular mechanism of glioma to predict prognosis and target gene therapy. The goal of this research was to discover the hub genes of glioma and investigate their prognostic and diagnostic usefulness. In this study, we collected mRNA expression profiles and clinical information from glioma patients in the TCGA, GTEx, GSE68848, and GSE4920 databases. WGCNA and differential expression analysis identified 170 DEGs in the collected datasets. GO and KEGG pathway analyses revealed that DEGs were mainly enriched in gliogenesis and extracellular matrix. LASSO was performed to construct prognostic signatures in the TCGA cohort, and 17 genes were used to build risk models and were validated in the CGGA database. The ROC curve confirmed the accuracy of the prognostic signature. Univariate and multivariate Cox regression analyses showed that all independent risk factors for glioma except gender. Next, we performed ssGSEA to demonstrate a high correlation between risk score and immunity. Subsequently, 7 hub genes were identified by the PPI network and found to have great drug targeting potential. Finally, RPL39, as one of the hub genes, was found to be closely related to the prognosis of glioma patients. Knockdown of RPL39 *in vitro* significantly inhibited the proliferation and migration of glioma cells, whereas overexpression of RPL39 had the opposite effect. And we found that knockdown of RPL39 inhibited the polarization and infiltration of M2 phenotype macrophages. In conclusion, our new prognosis-related model provides more potential therapeutic strategies for glioma patients.

## Introduction

Gliomas are the most common primary intracranial malignancies. According to the WHO, gliomas are classified into four grades, which define grades II and III as low-grade gliomas and grade IV as glioblastomas (1). Glioma treatment is standardized and includes intraoperative maximum resection and postoperative chemoradiotherapy (2). Although glioma therapy options have advanced significantly in recent decades, the treatment impact of glioma is far below expectations and faces significant obstacles because of its high heterogeneity and recurrence (3). As a result, there is an urgent need to investigate novel therapy techniques for glioma.

In studies of glioma genome analysis, it was demonstrated that genetic alterations in genes can play a crucial role in gliomagenesis by modifying essential signals and altering pathways for basic intracellular functions such as tumour cell development, proliferation, metastasis, and invasion (4). A large number of molecular biomarkers have been employed for the pathological diagnosis and prognostic evaluation of tumours. For example, the WHO classification of tumours of the central nervous system integrates isocitrate-mutated dehydrogenase (IDH) and codeletion of the short arm of chromosomes 1 and 19 of the long arm (1p/19q) (5). The new WHO classification guidelines make the diagnosis of glioma more accurate and the prognosis more relevant. Drugs targeting specific molecular biomarkers of glioma are also undergoing extensive clinical research. Terameprocol is a global transcriptional inhibitor that induces cell cycle arrest by selectively inhibiting specific protein 1 (Sp1), and it has been studied in stages I and II of recurrent high-grade glioma (6). In recurrent high-grade glioma, a phase II trial of nintedanib, a triple tyrosine kinase receptor inhibitor, is being conducted to overcome resistance to VEGF treatment (7). However, due to the high invasiveness and drug resistance of gliomas, most targeted drugs ultimately fail. As a result, the development of novel and effective biomarkers and their therapeutic targets is critical not only for glioma diagnosis and prognosis prediction but also for drug screening (8).

With the development of science and technology, microarray technology and bioinformatics have been widely used in tumour gene expression research, making molecular targeted therapy more precise and individualized (9). In this study, we used WCGA and differential expression analysis to identify DEGs in gliomas in public databases and then analysed their biological roles and enriched pathways. Then, we identified a 17-gene signature that provided the best prediction of overall survival. Finally, we selected the hub gene RPL39 for further *in vitro* experiments. Our results suggest that the development of a differentially expressed 17-gene-based risk score has potential value in predicting the prognosis of glioma patients.

## Data and methods

### Data collection

In this study, we collected a total of 4 public cohorts. Among them, the mRNA expression data and clinical information of glioma patients were obtained from the TCGA database (https://portal.gdc.cancer.gov/). The CGGA database (http://www.cgga.org.cn/) was used to validate the prognostic model. In addition, mRNA data from normal brain tissue were collected from GTEx (https://xenabrowser.net/datapages/). The GSE68848 (28 controls vs. 228 cancers) and GSE4920 (23 controls vs. 153 cancers) datasets in our study were downloaded from the GEO database (https://www.ncbi.nlm.nih.gov/geo/). As shown in **Table S1**, clinical information of all glioma patients was downloaded from the database. All data were searchable online. Finally, we used the R package “limma” of the “normalizeBetweenArrays” function to reduce batch effects that may exist between or within the four cohorts (10).

### Patient samples

This study was approved by the Ethics Committee of the Renmin Hospital of Wuhan University [approval number: 2012LKSZ (010) H]. None of the patients received radiotherapy or chemotherapy before surgery, and informed consent was obtained. Among the 37 clinical samples, 32 glioma samples included 17 low-grade gliomas and 15 high-grade gliomas, and 5 normal brain tissues were derived from patients with severe brain injury.

### Weighted correlation network analysis

We used the “WGCNA” package in R software to cluster genes with similar expression patterns in the TCGA+GTEx database. We set a minimum threshold and built a dendrogram. In the clustering results, genes with similar characteristics were assigned to the same module.

### Construction and validation of the risk signature

According to the survival status, survival time, and expression levels of related genes of glioma patients, least absolute shrinkage and selection operator (LASSO) regression analysis using the “glmnet” package was used to avoid overfitting. Then, the genes and their regression coefficients were obtained according to the most suitable λ value. Finally, we calculated each patient’s risk score according to the following model formula: Risk Score = $\sum_{\text{i}=1}^{n}\text{coef(i)}*x(i)$ , where n was the number of prognostic genes, Coef(i) was the regression coefficient, and x(i) was the expression level of the gene. All cases were classified into low- and high-risk groups based on the median risk score. The KM curve was used for survival analysis, the logrank test and Cox regression algorithm were used to assess the significance of differences in survival probability between the clinical features. The R package “survivalROC” was used to visualize time-dependent ROC curves and calculate AUC values to evaluate the prediction accuracy of the 17-gene signature. All heatmaps were visualized by the R package “pheatmap”.

### Analysis of tumour immunity and mutation

The XCELL, TIMER, QUANTISEQ, MCPCOUNTER, CIBERSORT, CIBERSORT-ABS, and EPIC algorithms were used to estimate immune infiltration in the low- and high-risk groups. Then, differential analyses of the stromal, immune, ESTIMATE scores, and immune cells were performed by the R software packages “estimate” and “ssGSEA” (11). The TIDE score for each glioma patient was determined by the online website (http://tide.dfci.harvard.edu/login/) (12). Finally, to assess the potential impact of risk score on the response to immunotherapy, we analysed associations between low- and high-risk groups with immune checkpoints and chemokines (13). Tumour mutational burden (TMB) was downloaded from the TCGA database and was defined as the numbers of insertions/deletions and substitution mutations per million bases, and the mutation profiles of low- and high-risk groups were analyzed by the R package “maftools” (14).

### Enrichment analysis of GO, KEGG, and GSEA

We used the R package “clusterProfile” to perform the GO, KEGG and GSEA enrichment analysis (30407594). For the GO and KEGG enrichment analysis, p value< 0.05 and FDR< 0.05 were defined as significantly different and the R package “ggplot2” was used to visualized the results. We obtained a subset of c5.go.V7.5.1.symbols.gmt from GSEA database (http://www.gsea-msigdb.org/gsea/downloads.jsp). Based on gene expression profiles and risk score, with a minimum gene set of 5 and a maximum gene set of 5000, p value< 0.05 and FDR< 0.25 were considered statistically significant.

### Protein−protein interaction network

In the STRING database (https://string-db.org/), a protein−protein interaction (PPI) network was constructed with a confidence level of 0.15. Cytoscape software (v3.7.2) was used to visualize the network (15). Finally, the plug-in MCODE of the Cytohhuba plugin was used to identify hub genes.

### Immunohistochemistry

Paraffin-embedded tissue sections were deparaffinized with xylene and rehydrated through a graded alcohol series. Heat-induced antigen retrieval was performed by immersing tissue sections in 10 mM citrate buffer (pH 6.0) for 10 min at 98°C. Subsequently, sections were blocked with serum for 30 min and incubated with primary antibody overnight at 4°C. The next day, sections were incubated with HRP-conjugated secondary antibody (Servicebio, China) for 1 h at room temperature. Sections were then stained with a DAB kit and counterstained with haematoxylin. Images were finally obtained by an Olympus BX51 microscope (Olympus, Japan), and tissue sections were independently investigated and scored by two researchers.

### Western blot analysis

RIPA lysate (Servicebio, China) was used to extract total protein from pretreated cells. Protein samples were electrophoresed on SDS-polyacrylamide gels and transferred to PVDF membranes. After blocking with skim milk for 1 h, the membrane was incubated with the corresponding primary antibody overnight at 4°C. RPL39 (14990-1-AP, 1:1000), CD206 (18704-1-AP, 1:1000), Arg1 (16001-1-AP, 1:1000) was purchased from Proteintech and tubulin (M20005, 1:3000) was purchased from Abmart. The membrane was then incubated with horseradish peroxidase (HRP)-conjugated secondary antibody for 1 h at room temperature and the ECL detection system (BIO RAD, USA) was used for visualization.

### Cell culture and induction and transfection

Human glioma cell lines (U251 and U87) and acute monocyte leukemia cell line (THP-1) were purchased from the Shanghai Institute of Biochemistry and Cell Biology (Shanghai, China). Glioma cells were cultured in DMEM (Dulbecco’s Modified Eagle’s Medium) supplemented with 10% foetal bovine serum (FBS) and THP-1 was grown in RPMI 1640 containing 15% FBS. These cells were cultured at 37°C and 5% CO_2_. For the induction of THP-1 cells, we first incubated THP-1 cells with 320 nM of PMA (Mce, China) for 24 h to induce M0 phenotype macrophages, and then added 20 ng/ml IL-4 and 20 ng/ml IL-13 for 24 h to obtain M2 phenotype macrophages. U87 cells were seeded in the lower chamber of a non-contact transwell and M2 macrophages were seeded in the upper chamber for 24 hours of co-culture. RPL39 siRNA and Flag plasmid were designed and purchased from RiboBio (Guangzhou, China). The sequences of siRNA for RPL39 were GUCACGAUCAUGUUACCAUTT (sense) and AUGGUAACAUGAU-CGUGACTT (antisense). Transient transfection with 2.0 µg plasmid per well in 6-well plates was performed with Lipofectamine 3000 (Invitrogen, USA) according to the manufacturer’s protocol. After 12-24 hours, these cells were collected for the following experiments.

### Cell proliferation assay

Cell Counting Kit-8 (CCK-8) assay: The cell suspension with a density of 5,000 cells was seeded in a 96-well plate for 24 h, and then CCK-8 solution was added to the corresponding wells at 0, 24, 48, and 72 h, respectively. The cells were incubated at 37°C for 1 hour, and finally, the optical density (OD) was read at 450 nm.

Colony formation: After transfection for 24 h, 500 cells were seeded into 6-well plates and cultured in complete medium for two weeks. When cell colonies were visible to the naked eye, the cells were fixed with paraformaldehyde for 20 minutes and stained with crystal violet. Finally, the cell colonies were imaged and counted.

Edu assay: The transfected cells were seeded on sterile glass slides in 6-well plates and cultured for 24 h. Following the manufacturer’s instructions, after incubating the cells with EdU reagent (C10310–1, RiboBio, Guangzhou, China) at 37°C for 1 h, the cells were fixed with 4% paraformaldehyde, and the nuclei were stained with DAPI. Photographs were finally captured under a fluorescence microscope.

### Wound healing and transwell assay

Transfected cells were seeded in 6-well plates. When the cell density reached 90%, a 200-µl sterile pipette tip was used to draw a scratch on the cell monolayer. After the cells were cultured in serum-free medium for 24 h, photographs were captured at the same position with an inverted microscope. In the Transwell assay, Transwell chambers (Corning, USA) precoated with Matrigel (R&D, USA) were used to analyse the invasive ability of cells. The transfected cells were seeded in serum-free medium in the upper chamber, and serum-containing medium was added to the lower chamber. After incubation at 37°C for 24 h, the cells were fixed and stained with crystal violet. Finally, the cells were counted under a microscope.

### RNA isolation and RT-PCR

Total cellular RNA was extracted using TRIzol reagent (Invitrogen, USA). PrimeScript RT kit (Takara, Japan) was used to synthesize cDNA and SYBR Green II Mixture kit (Takara, Japan) was used to perform qRT-PCR reactions on the ABI StepOne real-time PCR system (Applied Biosystems, USA) according to the reagent manufacturer’s instructions. The relative expression of mRNA was normalized by GAPDH and calculated by relative quantification (2^−ΔΔCt^). All primers were purchased from Sangon Biotech (Shanghai) and primer sequences were listed as follows: GAPDH primer (forward primer, GGGGCTCTCCAGAACATC; reverse primer, TGACACGTTGGCAGTGG); TNF-α primer (forward primer, CCCTCCTTCAGACACCCT; reverse primer, GGTTGCCAGCACTTCACT); iNOS primer (forward primer, CCTGGAAAACCCATGTCTG; reverse primer, GGGACGCCATTGTCTTG); CD206 primer (forward primer, GAAGCCAAGGTCCAGAAA; reverse primer, TGTTGAAAGC-GTATGTCCA); Arg1 primer (forward primer, GGAAGTGAACCCATCCCT; reverse primer, GATTACCCTCCCGAGCA).

### Statistical analysis

All data were analysed by R studio (version 4.2.0) and GraphPad Prism 9.0.0. Student’s t test was used to analyse the differences between the two groups. One-way analysis of variance (ANOVA) was used for comparisons between three or more groups. p< 0.05 was considered statistically significant.

## Results

### Identification of DEGs in collected datasets

**Figure 1:**
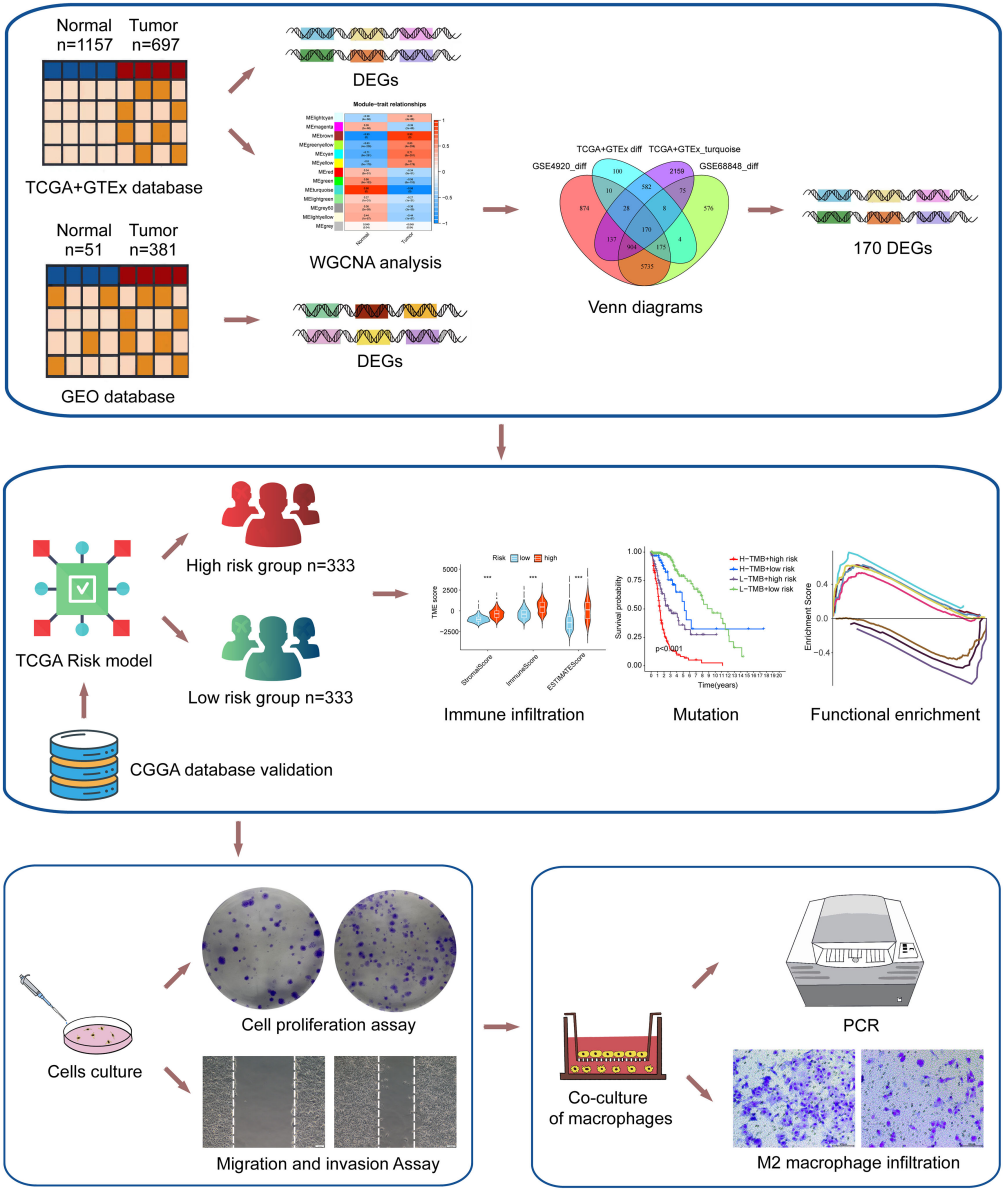
The flowchart of the study.

**Figure 2:**
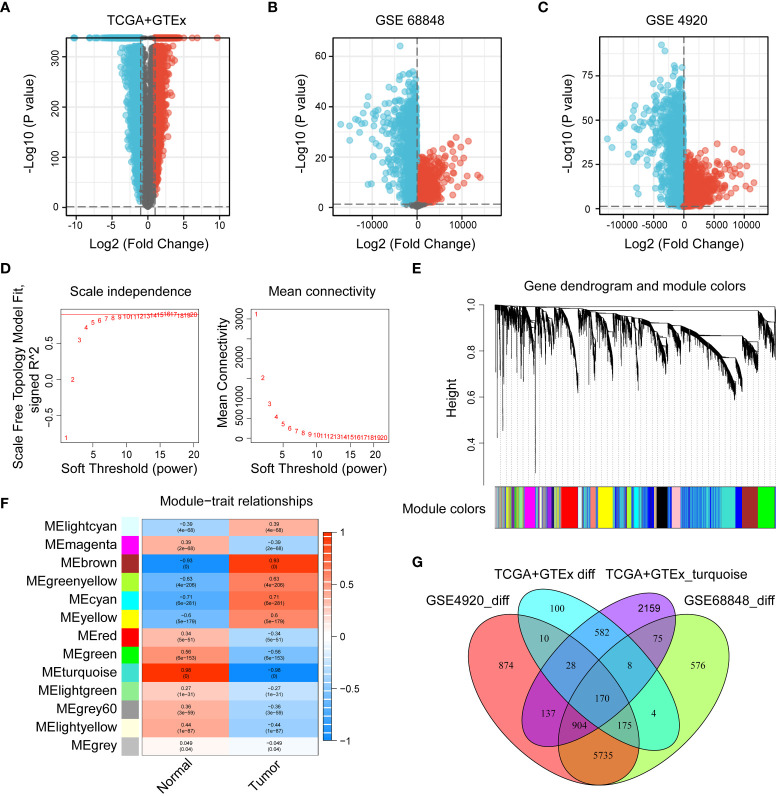
Identification of DEGs in the collected datasets. **(A–C)** Volcano diagrams of the TCGA+GTEx, GSE 68848, and GSE 4920 datasets. Red represents upregulated genes, black represents genes with no significant difference, and blue represents downregulated genes. **(D, E)** The selection of soft-thresholding power and construction of a scale-free network. **(E)** Cluster dendrogram in which similar genes are grouped into the same module. **(F)** Module-trait relationships, where the turquoise module has the highest correlation (cor = 0.96 and p = 0). **(G)** Venn diagram screening 170 DEGs in 4 glioma cohorts.

The flow chart of the study was shown in **Figure 1**. To investigate the gene expression profiles in gliomas, we collected 4 datasets and merged the TCGA and GTEx datasets to evaluate the gene expression differences between glioma and normal tissues. First, we performed differential expression analysis on the collected datasets. Under the conditions we set (abs of logFC>1 and adj. p value< 0.05), the TCGA+GTEx dataset included 1077 DEGs (**Figure 2A**), the GSE 68848 dataset included 7647 DEGs (**Figure 2B**) and the GSE 4920 dataset included 8033 DEGs (**Figure 2C**). Then, the WGCNA package in R was used to construct a coexpression network in the TCGA+GTEx dataset. Based on two evaluation criteria (scale independence and mean connectivity), the soft threshold was set at 8 (**Figures 2D, E**), and genes were divided into 13 modules. As shown in **Figure 2F**, the turquoise module had the highest correlation with gene characteristics and included 4063 genes. Then, we constructed Venn diagrams to identify 170 intersecting genes in the 4 screened gene sets (**Figure 2G**). Finally, GO and KEGG enrichment analysis indicated that these 170 genes were mainly related to cell development and extracellular matrix (**Figure S1A, B**). In short, these 170 genes may play important roles in glioma diagnosis and prognosis.

### Construction and verification of a 17-gene risk signature

**Figure 3:**
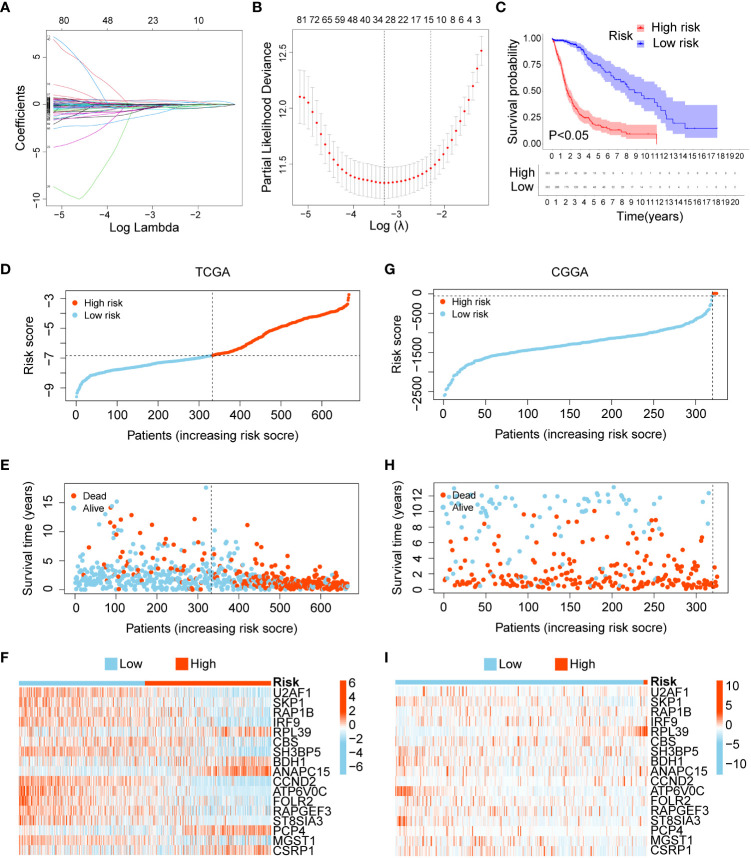
Construction and verification of a 17-gene risk signature. **(A)** LASSO coefficient profiles of the 170 DEGs. **(B)** Cross-validation for optimal parameter selection in the LASSO regression model. **(C)** Overall survival analysis between the low- and high-risk groups. **(D–F)** The risk score , survival status, and risk genes in the TCGA training set. **(G–I)** The risk score, survival status, and risk genes in the CGGA validation set.

To build a more efficient model to analyse clinical features and predict prognosis, LASSO regression analysis was performed on 170 DEGs, and 17 candidate genes with the most powerful predictive features were identified (U2AF1, SKP1, RAP1B, IRF9, RPL39, CBS, SH3BP5, BDH1, ANAPC15, CCND2, ATP6V0C, FOLR2, RAPGEF3, ST8SIA3, PCP4, MGST1, and CSRP1) (**Figures 3A, B**). Risk score were calculated based on the expression levels and matching coefficients of these 17 genes (risk score = U2AF1*(-0.2693) + SKP1*(-2.1119) + RAP1B*(-0.5732) + IRF9*(-0.9094) + RPL39*(0.51087) + CBS*(-0.3459) + SH3BP5*(-0.37226) + BDH1*(0.6100) + ANAPC15*(0.4592) + CCND2*(-0.3396) + ATP6V0C*(-3.1010) + FOLR2*(-1.0969) + RAPGEF3*(-1.4154) + ST8SIA3*(-1.6129) + PCP4*(0.3333) + MGST1*(-0.4300) + CSRP1*(0.3475)), and glioma patients were divided into low- and high-risk groups. The KM curve showed that the survival time of glioma patients in the high-risk group was significantly shorter than that in the low-risk group (**Figure 3C**). For the TCGA training set and the CGGA validation set, the risk score, survival status, and risk genes of glioma patients are shown in **Figures 3D–I**. In conclusion, a risk score based on the 17-gene signature was the most effective in predicting the prognosis of glioma patients.

### The risk score could be an independent factor for predicting the overall survival of glioma patients

**Figure 4:**
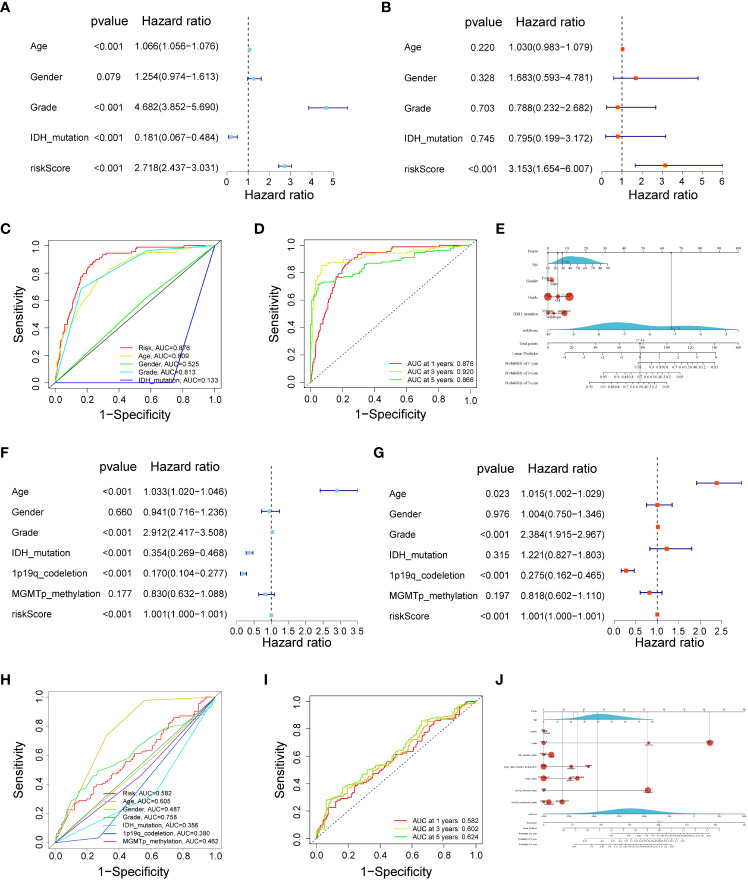
The risk score could be an independent factor for predicting the overall survival of glioma patients. **(A)** Univariate Cox analysis in the TCGA training cohort. **(B)** Multivariate Cox analysis in the TCGA training cohort. **(C)** ROC curve for the risk score in the TCGA training cohort. **(D)** Time-dependent ROC curves at 1, 3, and 5 years in the TCGA training cohort. **(E)** Nomograms were based on clinical features and risk score in the TCGA training cohort. **(F)** Univariate Cox analysis in the CGGA validation cohort. **(G)** Multivariate Cox analysis in the CGGA validation cohort. **(H)** ROC curve for the risk score in the CGGA validation cohort. **(I)** Time-dependent ROC curves at 1, 3, and 5 years in the CGGA validation cohort. **(J)** Nomograms were based on clinical features and risk score in CGGA validation cohort.

Univariate and multivariate Cox regression analyses indicated that a 17-gene signature-based risk score might serve as an independent predictor for glioma patients (**Figures 4A, B**). Next, the ROC curve showed that our risk score had the highest AUC value (0.876) when compared to other clinical features, showing the best predictive potential (**Figure 4C**). The AUC values for time-dependent OS at 1, 3, and 5 years were 0.876, 0.920, and 0.866, respectively (**Figure 4D**). In addition, to accurately predict the prognosis of glioma patients, we constructed a nomogram based on clinical features and risk score, as shown in **Figure 4E**, with the risk score being the most prominent factor and the model overall C-index value was 0.859 (95% CI: 0.839-0.878). Finally, we validated these results in the CGGA cohort (**Figures 4F, G**). The risk score had a higher AUC value (0.582) (**Figure 4H**), and the AUC values for time-dependent OS at 1, 3, and 5 years were 0.582, 0.602, and 0.624, respectively (**Figure 4I**). As shown in **Figure 4J**, we also constructed nomograms of risk score combined with other important parameters in the CGGA validation cohort (C-index = 0.782, 95% CI: 0.752-0.812).

### Tumour-infiltrating immune cell profiles

**Figure 5:**
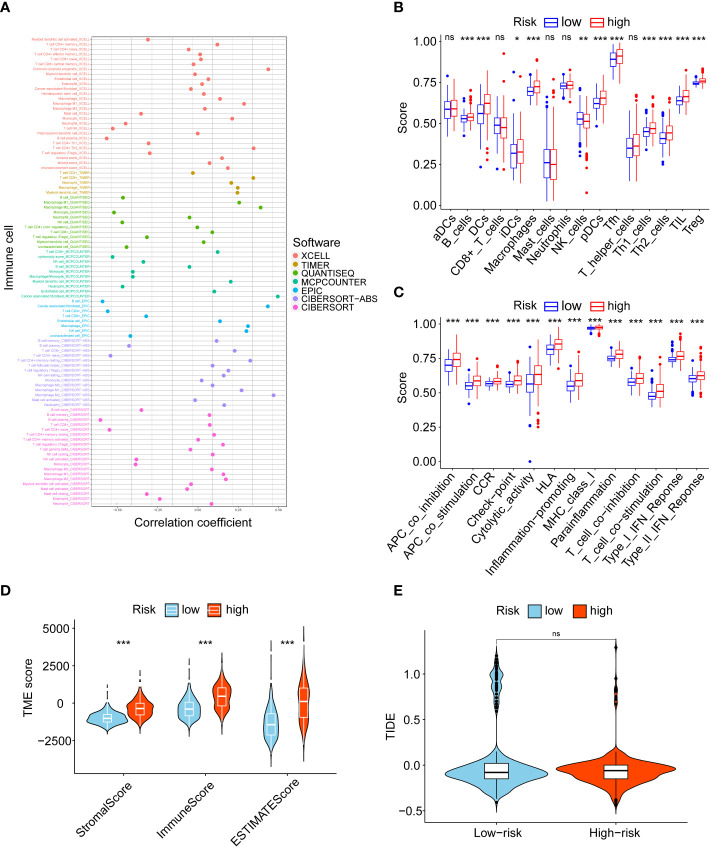
Tumour-infiltrating immune cell profiles. **(A)** Correlation between risk score and immune cells. **(B)** The analysis of 16 immune infiltrating cells between the low- and high-risk groups. **(C)** The analysis of immune functions between the low- and high-risk groups. **(D)** Comparison of the stromal, immune, and ESTIMATE scores between the low- and high-risk groups. **(E)** TIDE scores for low- and high-risk groups. ns, no significance, *P< 0.05, **P< 0.01, ***P< 0.001.

We assessed the association between risk score and immunity. First, we compared the correlation of risk score with different immune cells based on 7 algorithms, and the results showed that macrophages, cancer-associated fibroblasts, and CD4+ T cells were significantly positively correlated with risk score (**Figure 5A**). The ssGSEA algorithm was then used to analyse the infiltration statuses of 16 immune cell types in gliomas and their related functions in the low- and high-risk groups, and the box plots showed that the proportion of immune cells in the high-risk group was significantly higher than that in the low-risk group (**Figures 5B, C**). As shown in **Figure 5D**, the stromal, immune and ESTIMATE scores were significantly higher in the high-risk group than in the low-risk group. Finally, TIDE results showed no significant difference in immune escape between the low- and high-risk groups (**Figure 5E**). In conclusion, there was more immune cell infiltration in the high-risk group.

### Analysis of immune checkpoints, immune chemokines and immunotherapy

**Figure 6:**
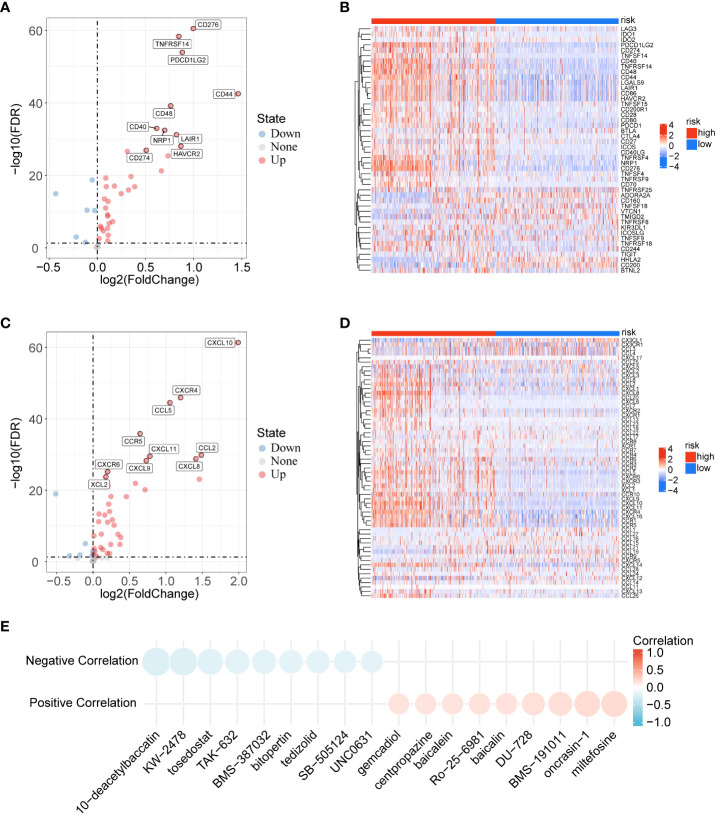
Analysis of immune checkpoints, immune chemokines and immunotherapy. **(A)** Volcano plot of the distribution of immune checkpoint-related genes in low- and high-risk groups. **(B)** Correlations between risk score and immune checkpoints in gliomas. **(C)** Volcano plot of the distribution of immune chemokines-related genes in low- and high-risk groups. **(D)** Correlations between risk score and immune chemokines in gliomas. **(E)** Correlation between risk score and drug sensitivity.

Immune checkpoints and chemokines play an important role in the regulation of immune cell function and are considered to be key factors affecting tumour immunotherapy. We explored whether immune checkpoints and chemokines differed between the low- and high-risk groups. The results showed that TNFRSF14, PDCD1LG2, CD44, and CD276 were significantly increased in the high-risk group in immune checkpoints (**Figures 6A, B**), while CXCL8, CXCL10, CCL2, and CXCR4 were significantly increased in the high-risk group in chemokines (**Figures 6C, D**). As shown in **Figure 6E**, we analyzed the association of risk score with drug sensitivity in the PRISM database (16). The results showed that 6 potential drugs (10−deacetylbaccatin, KW−2478, tosedostat, BMS−191011, oncrasin−1, miltefosine) were highly correlated with the risk score. In conclusion, the high-risk group might be more beneficial for immunotherapy.

### The association of risk score with mutational status

**Figure 7:**
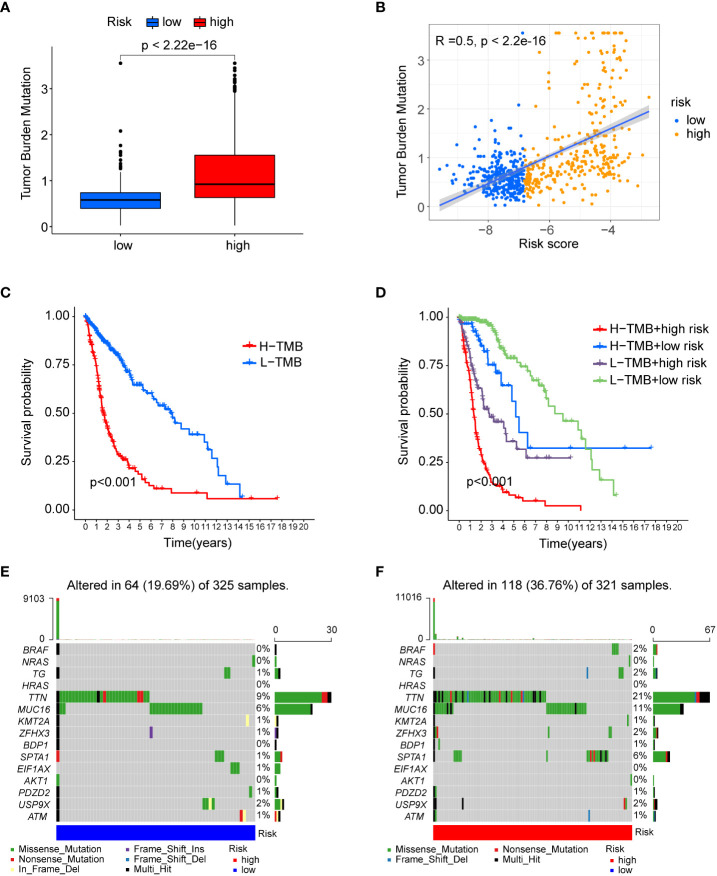
The association of risk score with mutational status. **(A)** Boxplots showing differences in TMB between the low- and high-risk groups. **(B)** Spearman’s correlation analysis showed a positive and significant correlation between TMB and risk score (R=0.5; P<0.001). **(C)** KM curve for low- and high-risk groups in the TCGA database. **(D)** KM curve of TMB combined with risk score. **(E)** Waterfall plot showing the top 15 highly mutated genes in the low-risk group. **(F)** Waterfall plot showing the top 15 highly mutated genes in the high-risk group.

Numerous studies have shown that tumour mutational burden (TMB) is another important factor affecting tumour immunotherapy (17). Here, we first compared TMB levels in the low- and high-risk groups, and as shown in **Figures 7A, B**, TMB was significantly elevated in the high-risk group and positively correlated with risk score. Then, the R package “survminer” was used to identify the best cut-off value to separate the patients into two clusters. K-M curve analysis showed that patients with high TMB had a shorter survival time than patients with low TMB (**Figure 7C**). Additionally, based on the link between TMB and risk score, we found that patients with high TMB and risk score had the worst prognosis (**Figure 7D**). Finally, the “maftools” R package was used to analyse and visualize somatic variation in risk subgroups. As shown in **Figures 7E, F**, the top 15 mutated genes were the same in the low- and high-risk groups, and TTN and MUC16 gene mutations were the most common. Taken together, the TMB and somatic variant rates were highly related to the prognostic-relevant risk score we constructed.

### Functional enrichment analyses

**Figure 8:**
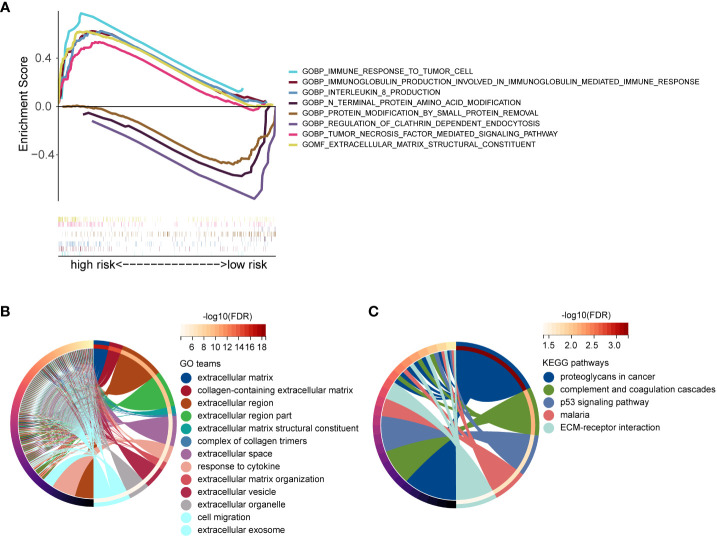
Functional enrichment analyses. **(A)** GSEA enrichment analysis of low- and high-risk groups. **(B)** GO functional enrichment analysis of DEGs between low- and high-risk groups. **(C)** KEGG pathway enrichment analysis of DEGs between low- and high-risk groups.

To elucidate the potential biological functions and pathways associated with these 17-gene signature. GSEA enrichment analysis showed that the high-risk group was mainly enriched in immune response and extracellular matrix, and the enrichment function of the low-risk group mainly included protein modification and pegulation of clathrin dependent endocytusis (**Figure 8A**). We identified 345 DEGs based on the expression data of the low- and high-risk groups (|Log2FC|>1 and p<0.05). Then, we performed a GO enrichment analysis, these 345 DEGs were mainly enriched in the extracellular matrix and cell migration (**Figure 8B**). As shown in **Figure 8C**, KEGG enrichment analysis indicated that these 345 DEGs were mainly related to the p53 signaling pathway and ECM-receptor interaction. Taken together, the 17-gene signature was mainly associated with immune response and the extracellular matrix.

### Drug sensitivity analysis of hub genes

**Figure 9:**
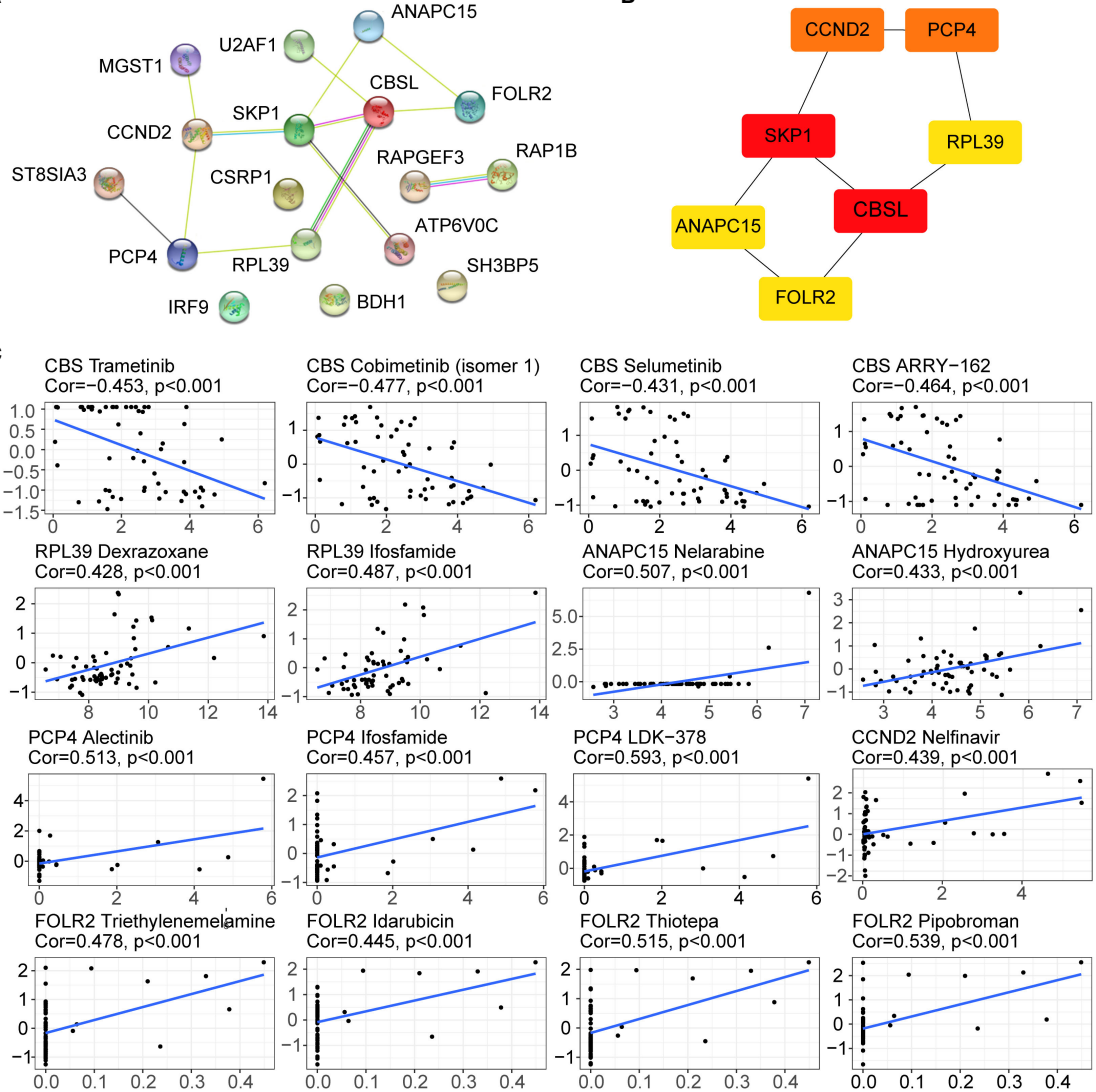
Drug sensitivity analysis of hub genes. **(A)** PPI network of the 17 genes. **(B)** Top 7 hub genes in the PPI network; greater scores in the network are represented by darker colours. **(C)** Drug sensitivity of the hub genes in the Cell Miner database.

In our study, the PPI network was used to analyse the interactions of 17 genes, including 17 nodes and 20 edges (**Figure 9A**). Meanwhile, the Cytohhuba plugin identified the top 7 core genes in the PPI network: CCND2, PCP4, SKP1, RPL39, CBSL, ANAPC15, and FOLR2 (**Figure 9B**). Next, the Cell Miner database was used to analyse the drug sensitivity of core genes. As shown in **Figure 9C**, the expression profiles of hub genes other than the CBS gene were positively correlated with drug sensitivity, which may be related to tumour resistance.

### RPL39 expression was upregulated in gliomas and predicted a worse prognosis for glioma patients

**Figure 10:**
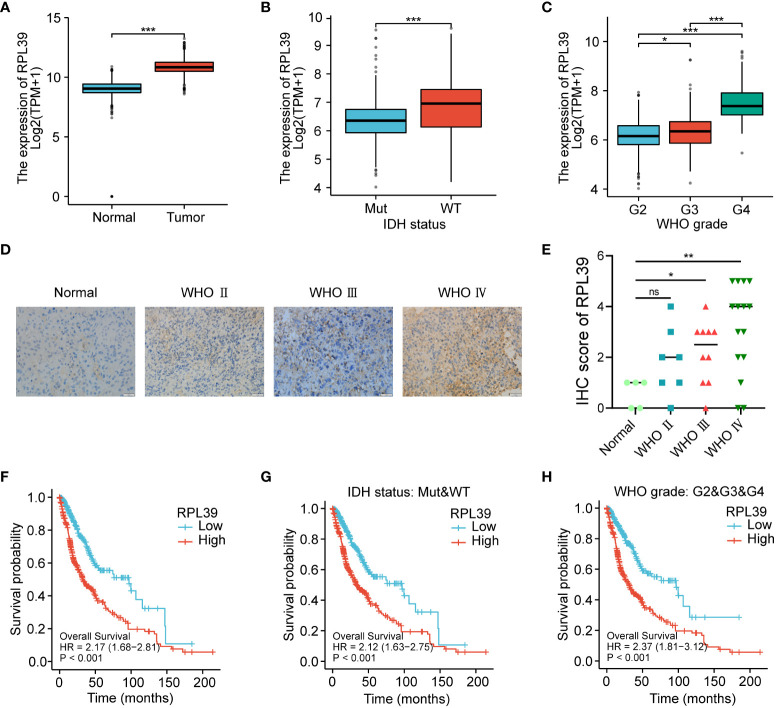
RPL39 expression was upregulated in gliomas and predicted a worse prognosis for glioma patients. **(A)** TCGA database analysis indicated that RPL39 mRNA expression is increased in glioma. **(B)** Correlations between RPL39 mRNA expression and IDH status. **(C)** Correlations between RPL39 mRNA expression and WHO grade. **(D, E)** Immunohistochemical staining of RPL39 in clinical samples, statistical analyses were shown on the right. **(F)** KM survival analysis for overall survival for glioma patients in TCGA database. **(G)** KM survival analysis of RPL39 mRNA expression compared with IDH status. **(H)** KM survival analysis of RPL39 mRNA expression compared with WHO grade. ns, no significance, *P< 0.05, **P< 0.01, ***P< 0.001.

To further explore the validity of the 17-gene signature, we selected one of the hub genes, RPL39, for *in vitro* validation. In the TCGA database, we found that the expression level of RPL39 in glioma tissue was significantly higher than that in normal brain tissue (**Figure 10A**). RPL39 was shown to be highly expressed in IDH wild-type glioma patients (**Figure 10B**), and its expression increased with glioma grade (**Figure 10C**). The results were consistent with the IHC staining of the clinical samples we collected (**Figures 10D, E**). In conclusion, RPL39 was overexpressed in gliomas and positively correlated with tumour aggressiveness. Next, the TCGA database was used to explore the effect of RPL39 on the survival of glioma patients, and the KM curve showed that the survival time of glioma patients with high RPL39 expression was significantly shorter than that of glioma patients with low RPL39 expression (**Figure 10F**). Furthermore, the expression of RPL39 was significantly associated with the prognosis of glioma patients with different clinical features (IDH status and WHO grade) (**Figures 10G, H**). In short, RPL39 is a novel prognostic marker in glioma.

### RPL39 promoted proliferation in glioma cells

**Figure 11:**
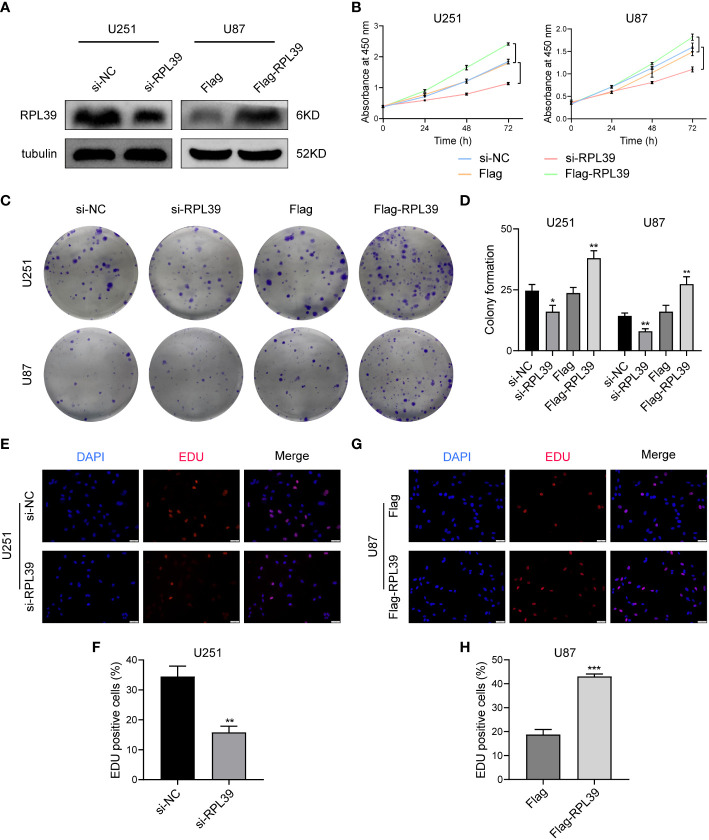
RPL39 promoted proliferation in glioma cells. **(A)** Western blot confirming successful plasmid construction. **(B)** A CCK8 assay was used to measure the viability of U251 and U87 cells. **(C, D)** Knockdown of RPL39 reduced colony numbers in U251 and U87 cells, whereas overexpression of RPL39 resulted in the opposite; statistics are shown on the right. **(E-H)** EdU assays were performed to assess cell proliferation. Red represents EdU-positive cells; blue represents nuclei. The percentage of EdU-positive cells was determined by counting erythrocytes/blue cells. *P< 0.05, **P< 0.01, ***P< 0.001.

Our study indicates that RPL39, as one of the hub genes related to cell development and extracellular matrix, is a potential oncogene. To explore the function of RPL39 in glioma, siRPL39 and Flag-RPL39 plasmids were used to specifically knock down and overexpress RPL39, respectively, and western blotting was performed to assess the efficacy of the plasmids (**Figure 11A**). First, the CCK8 test was performed to detect the viability of glioma cells, and the results showed that RPL39 knockdown significantly reduced the viability of U251 and U87 cells, while RPL39 overexpression increased cell viability (**Figure 11B**). Cell colony formation experiments showed that RPL39 knockdown greatly reduced the number of colonies in U251 and U87 cells, but RPL39 overexpression had the reverse effect (**Figures 11C, D**). Finally, an EdU assay was used to evaluate the proliferation ability of U251 and U87 cells, and EdU positivity indicated that the cells were in the division phase. The results showed that the number of positive cells in the RPL39 knockdown group was lower than that in the normal group, while the number of positive cells in the RPL39 overexpression group was higher than that in the normal group (**Figures 11E–H**). In conclusion, RPL39 could promote the proliferation of glioma cells.

### RPL39 promoted EMT in glioma cells

**Figure 12:**
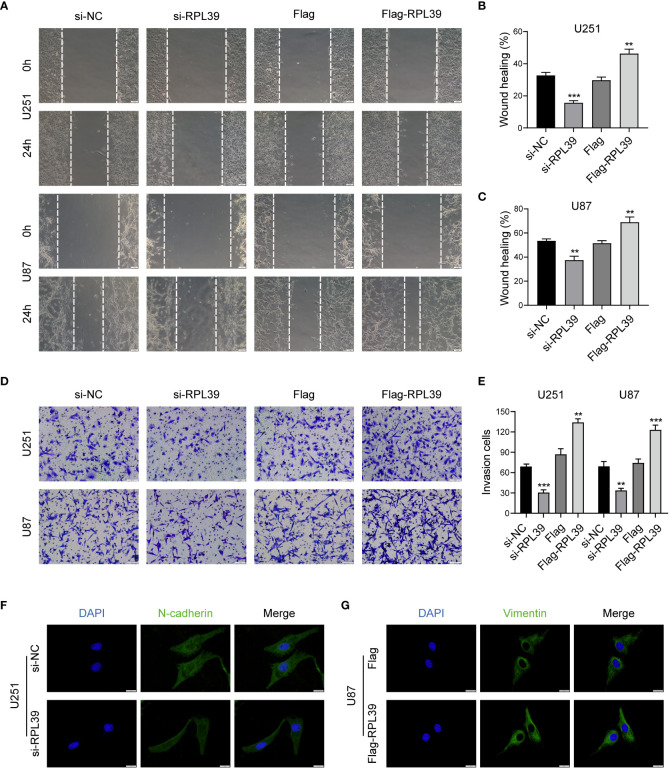
RPL39 promoted EMT in glioma cells. **(A-C)** Wound healing assays were performed to evaluate cell migration capacity. **(D, E)** Transwell assays were performed to evaluate cell invasion capacity. **(F, G)** Immunofluorescence was used to compare intracellular protein expression. **P< 0.01, ***P< 0.001.

In tumour research, disruption of the extracellular matrix is essential for tumour migration and invasion; therefore, we speculate that RPL39 may affect EMT progression in glioma cells. First, we tested the migratory ability of glioma cells by wound healing assay. As shown in **Figures 12A–C**, compared with the control group, the wounds in the RPL39 knockdown group healed slowly, while the spacing in the RPL39 overexpression group was significantly narrowed. Meanwhile, the Transwell assay showed that knockdown of RPL39 reduced the invasive ability of U251 and U87 cells (**Figures 12D, E**). Finally, to further explore the relationship between RPL39 and EMT, cellular immunofluorescence was used to detect the expression of key proteins in EMT. The results showed that N-cadherin expression was reduced in the RPL39 knockdown group, while Vimentin expression was increased in the RPL39 overexpression group (**Figures 12F, G** and **Figures S2A, B**). These results demonstrated that RPL39 promotes glioma cell migration and invasion.

### RPL39 induced M2 phenotype macrophages polarization and infiltration

**Figure 13:**
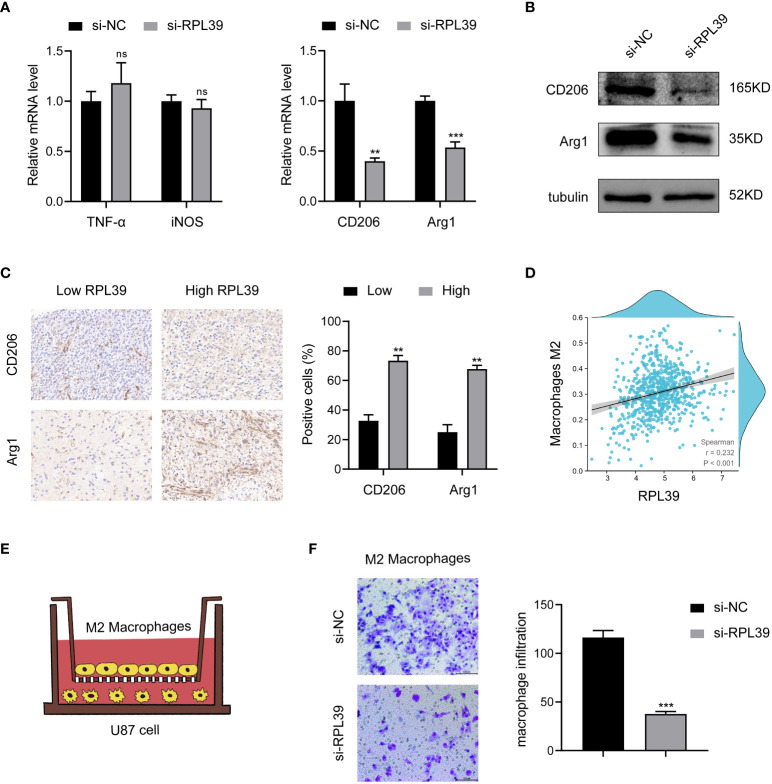
RPL39 induced M2 macrophages polarization and infiltration. **(A)** Marker mRNA expression levels in M1 phenotype macrophages and M2 phenotype macrophages. **(B)** Western blot analysis further analyzed the expression of M2 phenotype macrophage markers. **(C)** Immunohistochemical staining showed that RPL39 was positively correlated with the expression of CD206 and Arg1, statistical analyses were shown on the right. **(D)** RPL39 expression positively correlated with infiltration of M2 phenotype macrophages. **(E)** Schematic representation of co-culture of U87 cell and M2 phenotype macrophages. **(F)** Knockdown of RPL39 inhibited infiltration of M2 phenotype macrophages. ns, no significance, **P< 0.01, ***P< 0.001.

To further elucidate the correlation between RPL39 and tumor immunity. First, we collected conditioned medium of si-RPL39-transfected U87 cells and incubated with M0 macrophages for 24 h. The results of PCR showed that there was no significant change in the expression of M1 phenotype macrophages markers (TNF-α and iNOS), while the expression of M2 phenotype macrophages markers (CD206 and Arg1) decreased significantly (**Figure 13A**). At the same time, Western blot analysis showed that the protein levels of CD206 and Arg1 also decreased (**Figure 13B**). These results suggested that knockdown of RPL39 inhibited the polarization of M2 phenotype macrophages but had no effect on M1 phenotype macrophages. Next, we confirmed the positive correlation of RPL39 with the expression of CD206 and Arg1 in collected clinical samples and in public databases (**Figures 13C, D**). Finally, as shown in **Figure 13E**, we co-cultured U87 cells with M2 phenotype macrophages, and the results of transwell showed that knockdown of RPL39 significantly reduced the infiltration of M2 phenotype macrophages (**Figure 13F**). Briefly, RPL39 induced polarization and infiltration of M2 phenotype macrophages.

## Discussion

Gliomas are the most frequent malignant central nervous system tumours, accounting for more than 80% of all primary brain tumours (18). Because of glioma’s high heterogeneity and invasiveness, the 5-year survival rate of glioma patients remains very low (19), prompting people to aggressively investigate the pathophysiology and molecular targeted therapy of glioma. With the advent of the era of precision medicine and the development of bioinformatics, an increasing amount of research has focused on identifying markers or models that can accurately predict prognosis and provide individualized treatment for patients (20, 21). For example, Ma J et al. identified 5 prognosis-related markers in triple-negative breast cancer and provided potential therapeutic targets through multiomics and bioinformatics (22). Zhou L et al. developed an 8-gene signature to predict overall survival in colon cancer patients (23). However, similar research in gliomas was significantly less common than in other tumours, implying that more comprehensive and in-depth studies of gliomas are urgently needed.

In this study, we used multiple public datasets to investigate the predictive role of novel biomarkers in glioma. WGCNA and differential expression analysis were performed to identify 170 DEGs between glioma and normal tissues, and GO function and KEGG pathway enrichment analyses found that these genes were mainly associated with glioma progression and migration. LASSO regression and Cox regression analyses identified the best 17-gene signature, and the risk score of the 17-gene signature accurately predicted overall survival in glioma patients and could be considered an independent prognostic model. At the same time, the AUC value of the risk score reached 0.876 and the C-index value of the model was 0.859, which was much greater than the prognostic model constructed by the previous study, indicating that our 17-gene signature was more advantageous in precision (24–26). Aside from prognostic value, we found that the 17-gene signature was also related to tumour immunity and gene mutation. A growing number of studies have shown that tumour immunity is crucial for tumorigenesis and therapeutic response (27, 28). Yu M et al. found that high PARG expression was associated with poor prognosis in hepatocellular carcinoma, and knockdown of PARG enhanced the efficacy of immune checkpoint therapy (29). Abdelfattah N et al. identified S100A4 as an immunotherapy target and an independent prognostic factor in glioma patients by single-cell sequencing (30).

In previous studies, most of the 17 genes that constitute prognostic features have been shown to be associated with cancer progression, displaying a favourable or unfavourable prognostic significance in various tumours. S-phase kinase-associated protein 1 (SKP1) is a component of the SCF (SKP1/cullin-1/F-box) ubiquitin ligase complex. SCF complexes play important roles in cell division and cancer progression by degrading specific protein substrates through ubiquitination (31, 32). In colorectal cancer, SKP1 is highly expressed and is associated with poor patient prognosis (33). Purkinje cell protein 4 (PCP4), an anti-apoptotic peptide that binds calmodulin, has been shown to promote the migration and invasion of human breast cells (34) and to benefit the prognosis of lung adenocarcinoma patients (35). Folate receptor beta 2 (FOLR2) is one of the prognostic genes associated with the breast cancer tumour microenvironment (36), and patients with high FLOR2 expression in lung adenocarcinoma have longer overall survival (37). Ribosomal protein L39 (RPL39) is a component of the 60S ribosome on the X chromosome (XQ24) (38). In pancreatic cancer, knockdown of RPL39 inhibited cell proliferation and enhanced cell apoptosis (39). In metaplastic breast cancer, patients with high expression of RPL39 had lower overall survival, and RPL39 increased cell proliferation and migration by inducing nitric oxide synthase (INO)-mediated NO production (40). RPL39 has not been studied in glioma, and we further explored the function of RPL39 in glioma. As previously described, our *in vitro* results showed that RPL39 was highly expressed in gliomas and was associated with a worse prognosis. RPL39 promoted the proliferation of glioma cells and changed the EMT status of glioma cells; these results were consistent with those of previous studies.

In conclusion, we effectively constructed a predictive prognostic risk model based on a 17-gene signature using bioinformatics approaches such as WGCNA and LASSO regression. The model displayed strong predictive efficacy in both the training and validation cohorts, and clinical samples and *in vitro* studies confirmed the feasibility of the model. Despite meticulous planning, this experiment has some limitations, animal model experiments and potential mechanisms of action need to be further investigated.

## Conclusion

We comprehensively analysed the gene expression of glioma, and a novel 17-gene signature was constructed to maximize the prognostic assessment and molecular targeted therapy of glioma.

## Data availability statement

The original contributions presented in the study are included in the article/**Supplementary Material**. Further inquiries can be directed to the corresponding author.

## Ethics statement

The studies were reviewed and approved by the Institutional Ethics Committee of the Faculty of Medicine at Renmin Hospital of Wuhan University (approval number: 2012LKSZ(010)H). All patients signed informed consent forms.

## Author contributions

ST and MX contributed to data analysis and manuscript writing. LY, YX, and QS contributed to the review and revision of the manuscript. DT designed this study. All authors contributed to the article and approved the submitted version.

## Funding

This work was supported by the Hubei Province Health and Family Planning Scientific Research Project (WJ2017M019).

## Acknowledgments

We sincerely thank the central laboratory at the Renmin Hospital of Wuhan University.

## Conflict of interest

The authors declare that the research was conducted in the absence of any commercial or financial relationships that could be construed as a potential conflict of interest.

## Publisher’s note

All claims expressed in this article are solely those of the authors and do not necessarily represent those of their affiliated organizations, or those of the publisher, the editors and the reviewers. Any product that may be evaluated in this article, or claim that may be made by its manufacturer, is not guaranteed or endorsed by the publisher.

## Supplementary material

Supplementary Table 1 Baseline information of glioma patients.

## Abbreviations

TCGA, The Cancer Genome Atlas; GTEx, Genotype-Tissue Expression; CGGA, The Chinese Glioma Genome Atlas; DEGs, Differentially Expressed genes; GO, Gene Ontology; KEGG, Kyoto Encyclopedia of Genes and Genomes; BP, Biological Process; CC, Cellular Component; MF, Molecular Function; ROC, Receiver Operating Characteristic; AUC, Area Under the Curve; WHO, World Health Organization; OS, Overall Survival; KM, Kaplan−Meier; MCODE, Molecular Complex Detection; EMT, Epithelial-Mesenchymal Transition; ssGSEA, Single-sample Gene Set Enrichment Analysis; PMA, Phorbol 12-myristate 13-acetate.
