# Supplementary figures and images for: Identification and validation of a 17-gene signature to improve the survival prediction of gliomas
Source: Front Immunol. 2022 Sep 29;13:1000396. doi: 10.3389/fimmu.2022.1000396 (PMC9556650; doi:10.3389/fimmu.2022.1000396)

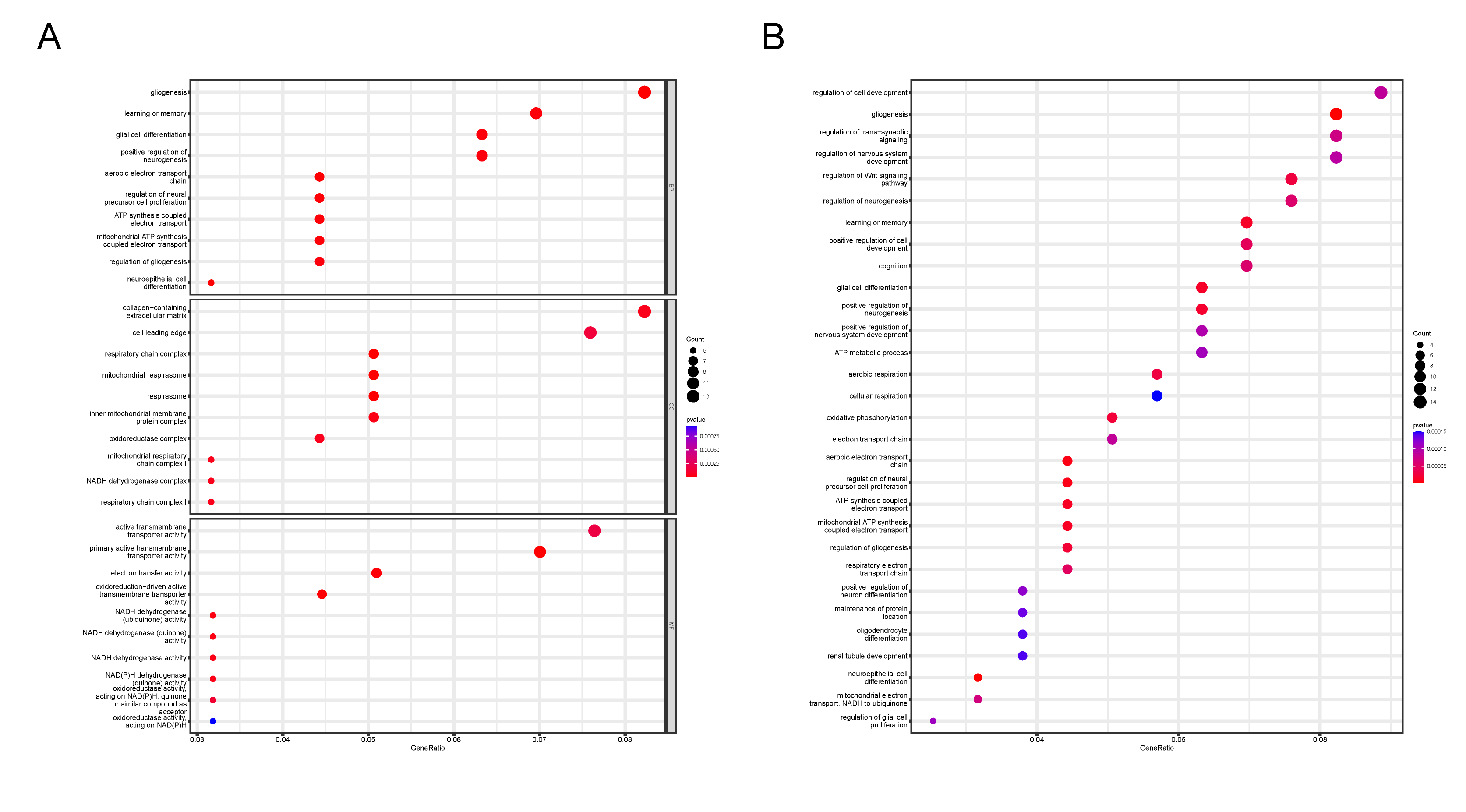

Supplement: Supplementary file 1 [file Image_1.tif]

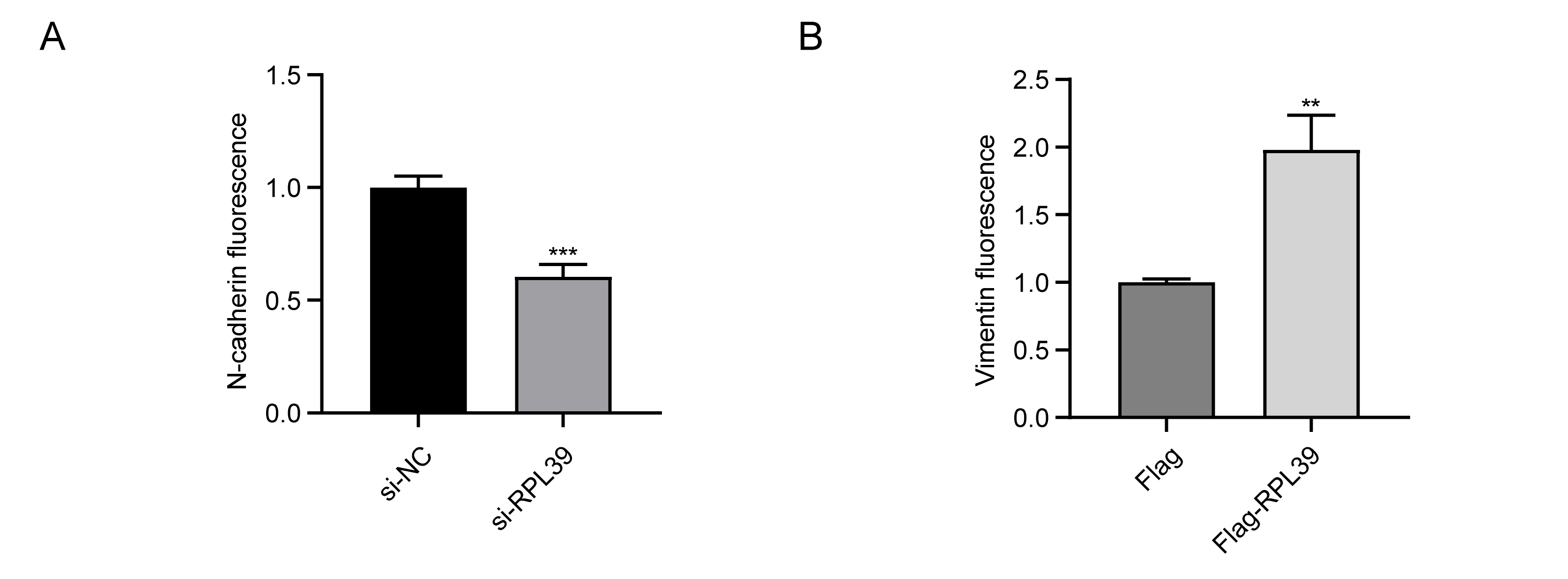

Supplement: Supplementary file 2 [file Image_2.tif]

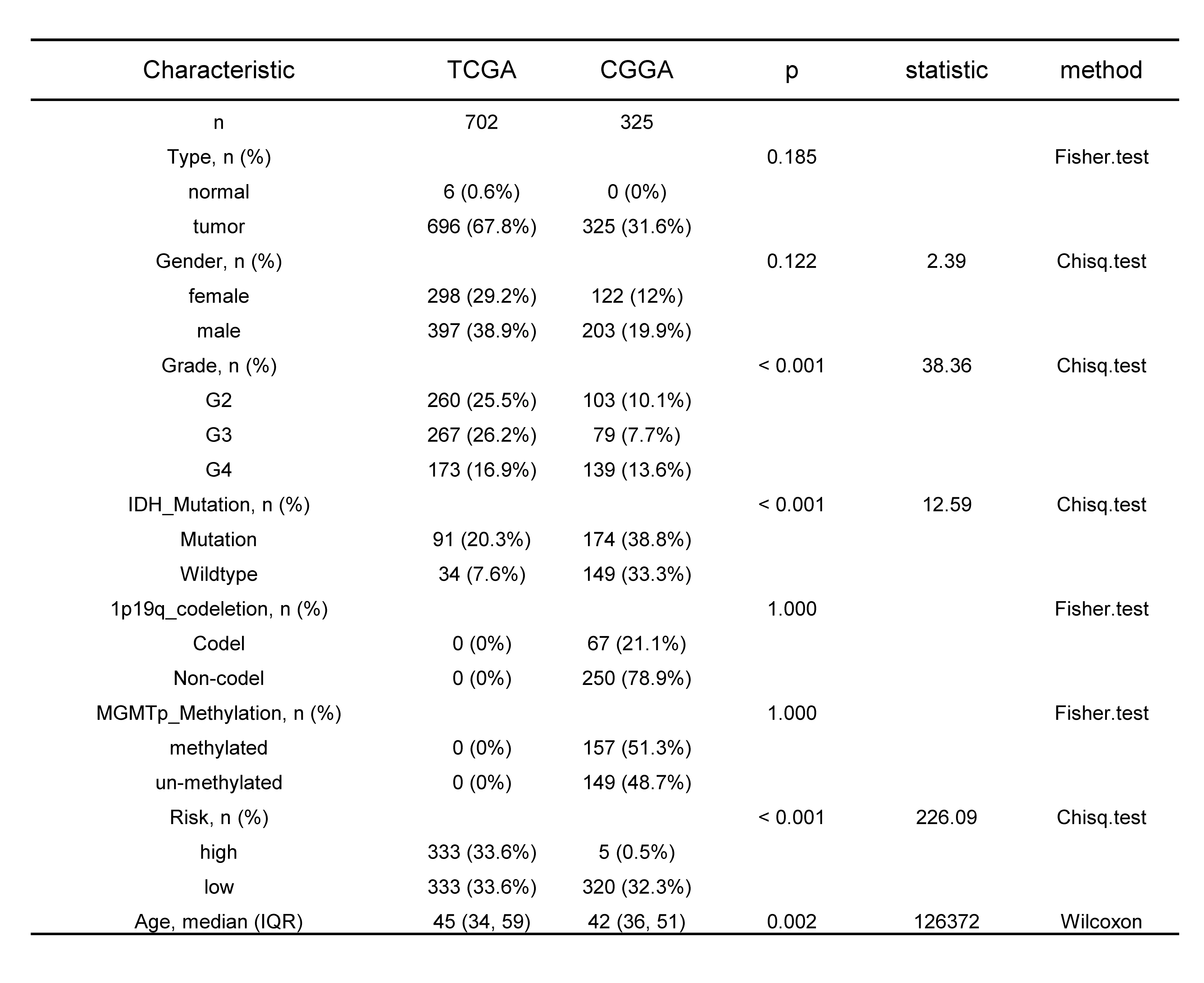

Supplement: Supplementary Table 1 — Baseline information of glioma patients. [file Image_3.tif]
